# Momentary Emotion Goals and Spontaneous Emotion Regulation in Daily Life: An Ecological Momentary Assessment Study of Desire for High Versus Low Arousal Positive Emotion

**DOI:** 10.1007/s42761-022-00108-7

**Published:** 2022-03-21

**Authors:** Benjamin A. Swerdlow, Devon B. Sandel, Jennifer G. Pearlstein, Sheri L. Johnson

**Affiliations:** 1 Department of Psychology, University of California, 2121 Berkeley Way West #1650, Berkeley, CA 94720 USA; 2 Dartmouth-Hitchcock Medical Center, Lebanon, NH USA; 3 University of Washington, Seattle, WA USA

**Keywords:** Emotion goals, Ideal affect, Emotion regulation, Ecological momentary assessment

## Abstract

Recent research has highlighted that emotion regulation strategy use varies both between and within people, and specific individual and contextual differences shape strategy use. Further, use of specific emotion regulation strategies relates to a wide array of differential outcomes, including mental health and behavior. Emotion goals (desire for a given emotion state) are thought to play a particularly important role in shaping people’s use of emotion regulation strategies; yet, surprisingly little is known about whether and how momentary emotion goals predict spontaneous strategy use in daily life. In the present investigation, we examined whether ideal desire for high versus low arousal positive affect was associated with subsequent use of specific emotion regulation strategies. Undergraduate participants (final *N* = 101) completed ecological momentary assessments (final *k*s = 1,932 for contemporaneous analyses, 1,386 for time-lagged analyses) of their momentary experienced affect, momentary desire for high versus low arousal positive affect, and emotion regulation. Desire for higher arousal predicted greater use of three disengagement strategies: distraction, expressive suppression, and experiential suppression. None of these strategies, though, were associated with sustained enhancement of high arousal (or low arousal) positive affect. These findings point to a possible disconnect between the strategies that people tend to use when they want to feel more arousal and the affective outcomes associated with use of those strategies.

Consider the following two scenarios. A surgical intern is feeling so excited to learn a new procedure that their hands are literally trembling. They need to reach a state of calm to ensure their hands will be steady, so they turn on soothing classical music and practice a breathing exercise. A kindergarten teacher wakes up on the first day of class feeling particularly sluggish, tired, and unmotivated. In order to have the energy to enthusiastically welcome a new class, they go for an early morning run while listening to up tempo pop music. These vignettes highlight the ways in which we often choose different emotion regulation strategies based on which emotion state we desire—in this case, calm vs. excited. In the present study, we examined how people’s emotion goals are associated with their subsequent emotion regulation strategy use.

Emotion regulation refers to processes that alter the timing, experience, or expression of emotion states, encompassing both automatic and deliberate processes (Gross, 2015). Empirical research has provided persuasive evidence of the differential consequences of specific emotion regulation strategies for a wide array of outcomes, such as mental health and relationship satisfaction (e.g., Aldao et al., 2010; Bloch et al., 2014). Contemporary theories and findings emphasize the importance of using strategies that are well-suited to the person, the situation, and the desired outcome (e.g., Aldao et al., 2015; Bonanno & Burton, 2013; Opitz et al., 2012; Troy et al., 2013; Wenzel et al., 2020). Understanding the factors contributing to emotion regulation strategy use has critical implications in clinical care, where people often need help adjusting or supplementing the repertoire of strategies they are using in a given context to achieve their goals.

In line with this, researchers have been increasingly interested in studying both spontaneous emotion regulation use in everyday life and emotion regulation choice in the lab over the past decade (e.g., Dixon-Gordon et al., 2015; Sheppes et al., 2011). That is, how and when do people regulate their emotions, and what factors shape these decisions? For example, experimental and ecological sampling studies have consistently shown that emotion type and emotion intensity shape emotion regulation strategy use and choice (e.g., Dixon-Gordon et al., 2015; Lennarz et al., 2019; Sheppes et al., 2011), among a wide array of other variables (see Young & Suri, 2020). Notably, people tend to choose disengagement strategies (e.g., avoidance, distraction), which require fewer cognitive resources, to regulate high intensity emotions, and more cognitively demanding engagement strategies (e.g., reappraisal, acceptance) when they are experiencing low intensity emotions (Sheppes et al., 2014). Of relevance to the present study, studies of the relationship between emotional intensity and emotion regulation strategy use have often collapsed valence and arousal into intensity.

One integral part of an emotion regulation decision is the emotion-related goal (e.g., Carver & Scheier, 2001; Fischer et al., 2004; Mauss & Tamir, 2014). A recent taxonomy of emotion regulation goals distinguishes between hedonic goals (the desire to change one’s immediate emotion experience) and instrumental goals (the desire to accomplish some other goal through the regulation of emotion; Tamir, 2016). Perhaps unsurprisingly, prior research has found that the hedonic goal of wanting to feel better may be the single most commonly endorsed emotion regulation goal in everyday life (e.g., Wilms et al., 2020). Consistent with this, many—but not all—of the strategies that people commonly use to regulate both negatively and positively valenced emotions are at least partially geared toward upregulating positive emotions.

Several recent investigations have provided empirical evidence that people’s momentary emotion goals are, in fact, associated with differential strategy use. For example, studies have shown that pro-hedonic goals (i.e., wanting to feel more positive and/or less negative) are positively associated with some specific emotion regulation strategies, such as reappraisal and distraction, but negatively associated with others, including suppression (Eldesouky & English, 2019; Wilms et al., 2020; see also Eldesouky & Gross, 2019). In a recent series of studies that also examined contra-hedonic goals (i.e., wanting to feel less positive and/or more negative), participants were more likely to select rumination when they wanted to up-regulate or maintain their negative emotions and distraction when they wanted to down-regulate their negative emotions (Millgram et al., 2019). In other words, the emotion regulation strategies that people use are shaped by how they want to feel—and, by extension, by the discrepancy between their current state and their desired state.

Within hedonic goals, past work has focused primarily on efforts to maintain or alter the valence of an emotional experience. At the same time, dimensional models of emotion emphasize that a combination of two (or more) neurophysiological systems underlie emotion states, specifically including valence (a spectrum of pleasant to unpleasant) and arousal (level of activation or alertness; Posner et al., 2005; Russell, 1980). As the vignettes above highlight, emotion regulation goals may sometimes center substantially or even primarily on arousal. In other words, there are times when people may not be wanting to feel better indiscriminately, but may instead be wanting specifically to feel calmer or more excited or may be wanting to move from a high arousal positive (HAP) state to a low arousal positive (LAP) state or vice versa; however, less empirical work has focused on emotion goals centered around changing arousal. The present study seeks to address this gap.

Clinical phenomena further highlight the importance of understanding which emotion regulation strategies people use to target arousal—and which are most successful at regulating arousal. Many clinical concerns have symptoms that are characterized by arousal dysregulation (e.g., panic, mania, post-traumatic stress disorder). Accordingly, interventions are often aimed not only at changing the valence of emotions, but also at regulating arousal (e.g., relaxation training to reduce over-arousal). For example, one recently developed intervention for bipolar I disorder teaches skills specifically to increase and maintain LAP—but not HAP—in light of evidence that an excess of HAP can be associated with negative consequences for people with bipolar disorder (Painter et al., 2019). Health psychology research likewise highlights how LAP and HAP can have differential consequences, such as LAP being associated with lower inflammation in cancer survivors (Moreno et al., 2016). In line with these observations, the present study focuses on how one’s desire for high versus low arousal *positive* emotion relates to emotion regulation strategy use. The desire for negative emotion, on the other hand, was not assessed, in part due to the expectation that people would rarely endorse wanting to feel more low arousal negative affect (LAN; e.g., bored) or high arousal negative affect (HAN; e.g., jittery).^1^

In considering how arousal goals relate to emotion regulation, it is important to consider that people differ in the affective states they ideally prefer to experience, referred to as their “ideal affect” (Tsai, 2007). Congruent with our focus on LAP and HAP, particularly robust cultural and individual differences have been documented in studies of ideal affect for preferences for LAP versus HAP. A large body of research has, moreover, demonstrated the consequences of these preferences for people’s affective experience, behavior, and decision-making (e.g., Chim et al., 2018; Tsai, 2007; Tsai, 2017; Scheibe et al., 2013; Sims et al., 2014). In one study, for example, participants higher in ideal LAP were more likely to choose to listen to a calmer (vs. more exciting) piece of music, and the reverse was true for participants higher in ideal HAP—evidence that situation selection is shaped by these affective preferences (findings summarized in Tsai, 2007). At the same time, recent empirical studies have documented considerable between- *and* within-person variation in emotion regulation strategy use (e.g., Catterson et al., 2017; McMahon & Naragon-Gainey, 2019; Southward & Cheavens, 2020), suggesting that while people do display individual differences in emotion regulation, strategy use is also sensitive to contextual demands. Given this, we used a within-person repeated measures approach to capture how arousal goals predict regulation behavior moment-to-moment rather than across people.

In summary, within the broader umbrella of hedonic emotion regulation goals, people may often want either more or less arousal in any given moment compared to how they currently feel. In turn, these goals may be associated with differential emotion regulation strategy use as people seek to attain desired, positively valenced affective states. Therefore, in the current study, we investigated time-lagged associations between desiring more high versus low arousal positive affect and emotion regulation strategy use. To complement these analyses, we also examined time-lagged associations between emotion regulation strategy use and experienced affect. That is, we tested two parts of the temporal process of emotion regulation in daily life: first, whether the desire for high or low arousal predicted specific emotion regulation strategy use, and second, how emotion regulation strategies impacted arousal. Consistent with recent calls to expand emotion regulation research to encompass a broader range of strategies (e.g., Ford et al., 2019), we included 9 specific emotion regulation strategies.

We did not make *a priori* predictions about each of the nine strategies, but we did make predictions about the two strategies for which there was the most agreement among the authors—those being relaxation and distraction. More specifically, we predicted that participants would be more likely to use relaxation strategies when they wanted to feel less arousal (and that relaxation would decrease arousal) given these strategies are capable of directly and rapidly targeting manifestations of physiological high arousal, like shallow breathing and muscle tension (e.g., Conrad & Roth, 2007; Hopper et al., 2019). Conversely, while empirical work on the upregulation of HAP is more limited, we predicted that participants would be particularly inclined to use distraction when they wanted to feel more arousal (and distraction would increase arousal), given that this category included behaviors such as going for a run that are used clinically to increase activation (cf. Hopko et al., 2003). We did not make specific predictions about how the remaining strategies would be related to desire for high vs. low arousal positive emotion, although we did expect that use of strategies that have been regarded as commonly maladaptive (e.g., rumination, experiential suppression, expressive suppression) would broadly predict increases in experienced negative affect/decreases in experienced positive affect and that use of strategies that have been regarded as commonly adaptive (e.g., acceptance, cognitive reappraisal, planning) would predict increases in experienced positive affect/decreases in experienced negative affect (cf. Aldao et al., 2010).

## Method

The present data were collected as part of a broader study investigating emotion, emotion regulation, and risky behavior in daily life.

### Participants and Procedures

A total of 881 potential participants from an undergraduate research participation pool at a large public university in California completed a brief pre-study screener. They were asked how often they had engaged in a variety of risky behaviors that are commonly associated with emotion dysregulation (non-suicidal self-injury, binge eating, purging, reckless driving, running on roofs, getting drunk, driving while intoxicated, illegal drug use, prescription drug use to get high, uncontrolled anger or aggression, and risky sex) in the past week on a 5-point ordinal scale (*not at all, once, 2–3 times, 4–6 times*, and *daily or more often*)*.* Guided by the objectives of the broader study, potential participants were invited to take part in the full study if they engaged in any of these behaviors in the past week at the following frequencies: self-harm 1+ times, got drunk 4+ times, or any of the other behaviors 2+ times. Of the 275 invited to participate, 122 participants enrolled, and 111 (91% of 122) completed the study. Participants received credit in their psychology courses in exchange for participation. We subsequently excluded data from one participant who answered over half (4 of 7) of baseline attention check items (e.g., *Please select “Almost never” for this question*) incorrectly, resulting in 110 participants with full data. On average, these 110 participants completed 18.11 (SD = 6.52) EMA surveys, or approximately 75% of all possible surveys. Nine participants were excluded from all analyses because their responses did not provide any pairs of complete back-to-back surveys, meaning that their responses could not be included in our core time-lagged analyses (see Data Analysis below). This final sample (*N* = 101) collectively completed a total of 1,932 surveys.

Participants in the final sample identified as cis female (76.9%), cis male (22.2%), and genderqueer or gender non-conforming (0.9%). Participants identified their ethnicities as: Asian/Asian American (43.5%), Black/African American (3.7%), Native Hawaiian/Pacific Islander (0.9%), White (27.8%), Other/Multiple Ethnicities (15.7%), or declined to answer (8.3%). 18.5% of participants identified as Hispanic or Latinx. The average age of the sample was 20.3 years (range 18 to 30). The modal participant placed themselves somewhat above the midpoint on the MacArthur Ladder of Subjective Socioeconomic Status (Mode = 7, Mean = 5.97, SD = 1.77; Operario et al., 2004).

The full study included baseline questionnaires completed on Qualtrics (not analyzed in the present study), followed by EMA surveys. Starting on the Thursday after completing the baseline, participants received 6 surveys each day for 4 days on their smartphones using the RealLifeExp app. Surveys were timed quasi-randomly between the hours of 10:00am and 11:59pm so that surveys were at least 110 min apart. Participants had 90 min to respond to each survey, and one reminder notification was sent if the survey was not completed within the first 45 min. On average, surveys took less than 2 min to complete.

### Measures

For each EMA survey, participants reported their momentary affective experience, momentary desire for high vs. low arousal positive emotion, and recent (i.e., since the past timepoint) emotion regulation strategy use.

#### Momentary Experienced Affect

To assess momentary affect, participants were asked in each survey to *Please rate each of the following emotions based on how you are CURRENTLY feeling* for a series of specific emotion items, each rated on a 7-point range slider from *Not at all* to *Extremely.* To reduce the total number of items, participants rated word pairs consisting of near neighbors (e.g., content and satisfied). We then used pairs of these ratings to form sum score composites (possible composite range = 2–14) for each of the four quadrants of the affective circumplex based on prior theory and research (cf. Feldman-Barrett, 2004). More specifically, LAP was formed from *Content/Satisfied* + *Calm/Peaceful* (repeated measures *r* = .46); HAP from *Proud/Confident* + *Enthusiastic/Excited* (repeated measures *r* = .46); LAN was from *Dull/Sluggish* + *Sad/Unhappy* (repeated measures *r* = .30); and HAN from *Afraid/Scared* + *Angry/Frustrated* (repeated measures *r* = .30). These composites were, themselves, moderately inter-correlated (repeated measures *r*s ranging from |.30| to |.53|) as shown in Table 1.

**Table 1:** Repeated measures correlations, intraclass correlations, means, and standard deviations for key predictor variables (N = 101; k = 1,932)

	(1)	(2)	(3)	(4)	(5)	ICC [95% CI]	M	SD
(1) Desire for arousal	--	−.08***	−.11***	.16***	.02	.22 [.16, .27]	3.59	.51
(2) Experienced HAP		--	.53***	−.46***	−.30***	.45 [.37, .53]	6.79	2.14
(3) Experienced LAP			--	−.30***	−.48***	.41 [.33, .47]	7.78	1.92
(4) Experienced LAN				--	.46***	.47 [.38, .54]	5.96	2.27
(5) Experienced HAN					--	.50 [.42, .57]	4.55	2.09

*Note*. *** *p* < .001. Data were aggregated within-participants prior to calculation of means and standard deviations

#### Momentary Desire for High Vs. Low Arousal Positive Emotion

Momentary desire for high vs. low arousal positive emotion was measured with one item: *Compared to how I am currently feeling, I would ideally like to feel…* with 5 response options: *Much more aroused/higher energy, Somewhat more aroused/higher energy, Neither higher nor lower energy, Somewhat calmer/lower energy,* and *Much calmer/lower energy.* Responses were coded on a 5 to 1 scale such that larger values represented desire for greater arousal.

#### Emotion Regulation Strategy Use

To assess recent emotion regulation strategy use, participants were asked: *Since the last timepoint, which of these things did you do to manage your emotions or change how you were feeling? Please select all that apply.* Participants had 9 response options related to specific emotion regulation strategies (adapted from Heiy & Cheavens, 2014) and *none of the above*. The response options were: *found an activity to keep yourself busy and distracted* (Distraction); *thought over and over again about the situation or your feelings* (Rumination); *controlled your feelings by not expressing them* (Expressive Suppression); *ignored or suppressed your feelings* (Experiential Suppression); *accepted your situation and/or your feelings* (Acceptance); *made a plan to make your situation better* (Planning); *thought about your situation in a different way* (Cognitive Reappraisal); *received emotional support from another person* (Social); and *engaged in a pleasant or relaxing activity* (Relaxation).

### Data Analysis

As preliminary analyses, we examined the univariate distributions of and bivariate associations between each of our key variables. Given the nested (within-person) data structure, we computed repeated measures correlations (i.e., the common within-person correlation; Bakdash & Marusich, 2017) to examine the structure of bivariate associations and intra-class correlations to evaluate the proportions of between- and within-person variance. Considering that our momentary experienced affect composites were inter-correlated, we computed variance inflation factors (VIF) to diagnose potential problems with multicollinearity (Zuur et al., 2010).

Then, we turned our attention to our core study aims. To examine temporal associations within our data, we created time-lagged variables. To keep the time intervals reasonably consistent, these time-lagged variables were only calculated for back-to-back surveys completed within a single day. This constraint reduced our effective sample for these analyses to *k* = 1,386. Our first aim was to ascertain the degree to which the likelihood of specific emotion regulation strategies could be predicted from desired high vs. low arousal positive emotion measured at the prior timepoint, adjusting for experienced affect. To test this aim, we computed nine parallel random intercept generalized mixed effects models using the logit link function. Strategy use (0 = No, 1 = Yes) was entered as the DV. Momentary desired high vs. low arousal positive emotion and experienced HAP, LAP, LAN, and HAN were entered as concurrent IVs. Variables were centered and standardized within-person prior to these analyses, consistent with our focus on estimating temporal associations among level-1 variables (Wang et al., 2019).

Our second, complementary aim was to evaluate the association between strategy use and change-over-time in experienced affect. To test this aim, we computed four parallel random-intercept linear mixed effects models—one each for experienced HAP, LAP, LAN, and HAN. In each model, experienced affect at time *T* was entered as the DV and the specific emotion regulation strategies were entered as the IVs alongside the corresponding experienced affect at time *T*−1.

All analyses were conducted in R v. 4.0.2 (R Core Team, 2020). Repeated measures correlations were implemented with the rmcorr package (Bakdash & Marusich, 2017). Intraclass correlations were calculated using the sjstats package (Lüdecke, 2021). Variance inflations factors were calculated using the performance package (Lüdecke et al., 2021). Mixed effects models were computed with the lme4 package using restricted maximum likelihood (REML) estimation (Bates et al., 2015). *p* values were estimated with the lmertest and pbkrtest packages using the Kenward-Roger approximation (Halekoh & Højsgaard, 2014; Kenward & Rogers, 1997; Kuznetsova et al., 2017). Pseudo-*r*-squared values were computed for the mixed effects models with the MuMIn package (Bartoń, 2009; Nakagawa & Schielzeth, 2013).

## Results

### Preliminary Analyses

All of the continuous variables approximated a normal distribution as established through visual inspection and calculation of skewness and kurtosis (Kline, 2011). Notwithstanding significant and expected bivariate repeated measures correlations between key IVs, model diagnostic tests indicated that variance inflation was tolerable (VIFs for all regression models < 2; Zuur et al., 2010). Momentary desire for higher vs. lower arousal was, moreover, only loosely correlated with momentary experienced affect (repeated measures *r*s ≤ .16), as shown in Table 1.

Some emotion regulation strategies were more commonly employed than others, with distraction (650 instances) and relaxation (579 instances) being the two most commonly endorsed strategies and expressive suppression (181 instances) and cognitive reappraisal (129 instances) being the two least commonly endorsed strategies. As shown in Table 2, significant repeated measures correlations were observed in emotion regulation strategy use. These correlations varied considerably in magnitude, with some strategies being quite robustly correlated (e.g., *r* = .57 for experiential suppression and expressive suppression) and other strategies being essentially unrelated to one another (e.g., *r* = −.02 for experiential suppression and planning). The overwhelming majority of these correlations were positive, suggesting that participants were frequently using multiple strategies within a single measurement interval—consistent with polyregulation.

**Table 2:** Repeated measures correlations between emotion regulation strategies (N = 101; k = 1,932)

	(1)	(2)	(3)	(4)	(5)	(6)	(7)	(8)	(9)
(1) Acceptance	--	.46***	.23***	.22***	.08	.35***	.20***	.14***	.28***
(2) Cognitive reappraisal		--	.10	.38***	.20**	.25***	.34***	.07	.45***
(3) Distraction			--	.13*	.05	.23***	.15**	.19***	.10
(4) Emotional support				--	.10	.17*	.21***	.15**	.24***
(5) Experiential suppression					--	.57***	−.02	−.14**	.27***
(6) Expressive suppression						--	.17*	.06	.41***
(7) Planning							--	−.03	.32***
(8) Relaxation								--	−07
(9) Rumination									--

*Note*. * *p* < .05; ** *p* < .01; *** *p* < .001

We also considered whether time-in-study was related to any of our affective variables. Small, significant associations between survey number and ratings of HAP and HAN were observed (*b*s = −.07 and −.04, *p*s = .001 and .03, respectively); however, time-in-study accounted for less than 1% of the variance in either of these (marginal pseudo-*r*-squareds = .005 and .001, respectively). Associations between survey number and ratings of LAN, LAP, and desire for high vs. low arousal positive emotion were non-significant (*p*s > .07). Comparable effects were observed when time-in-study was operationalized as day in the study (which doubled as day of the week) rather than survey number.

Intraclass correlations were variable for the measures of momentary experienced affect and desire for high vs. low arousal positive emotion, ranging from .22 (desire for high vs. low arousal) to .50 (momentary experienced HAN), as shown in Table 1. Intraclass correlations for emotion regulation strategy use were comparable, ranging from .25 (relaxation) to .44 (acceptance). These intraclass correlations are consistent with the presence of both individual and contextual influences on participants’ ratings of these affective variables.

One potentially important contextual influence may be circadian effects that organize the sleep-wake cycle. As a modest test for the presence of circadian organization in our affective variables, we computed five parallel random-intercept mixed effects models (DVs = momentary desired for high vs. low arousal, momentary experienced HAP, momentary experienced LAP, momentary experienced LAN, momentary experienced HAN). These were three-level models: measurement occasions were nested in days, which were nested in individuals. In each model, first- (linear), second- (quadratic), and third-order (cubic) orthogonal polynomials of time of day were entered as simultaneous IVs. We observed some evidence for time-of-day effects in momentary desire for high vs. low arousal positive emotion, in that significant associations with the first- (*b* = −3.37, *p* < .001), second- (*b* = −8.93, *p* < .001), and third- (*b* = −2.86, *p* = .001) order polynomials were statistically significant; however, these time-of-day effects collectively accounted for only a small proportion of the total variance in momentary desire for high vs. low arousal (marginal pseudo-*r*-squared = .05). With respect to momentary experienced affect, the only association that was observed to be significant was that between momentary experienced HAP and the third-order polynomial (*b* = −1.59, *p* = .02); as before, however, this association explained little of the overall variance (marginal pseudo-*r*-squared = .002). Given that time-of-day accounted for little variance, it was not included in tests of hypotheses.

As a final preliminary analysis, we examined whether desire for high vs. low arousal (at *T*−1) predicted experienced affect at the next timepoint (*T*), adjusting for experienced affect at the prior timepoint (*T*−1) to examine change. To do this, we computed four parallel random-intercept mixed effects models—one each for HAP, LAP, LAN, and HAN. Consistent with our focus on positively valenced affective states, desire for higher arousal did not significantly predict change in HAN (*b* = .03, *p* = .18) or LAN (*b* = −.02, *p* = .29). Somewhat surprisingly, desire for higher arousal also did not significantly predict change in HAP (*b* = .04, *p* = .06). As one would expect, desire for higher arousal negatively predicted LAP, although the magnitude of the effect was comparable to that of HAP (*b* = −.05, *p* = .04).

### Predicting Strategy Use from Desired High Vs. Low Arousal Positive Emotion

We first examined the zero-order repeated measures correlations between desired high vs. low arousal (measured at *T*−1), experienced affect (measured at *T*−1), and emotion regulation strategy use since the last timepoint (measured at *T*). As shown in Table 3, desire for higher arousal was associated with greater use of distraction, experiential suppression, and expressive suppression. No other significant associations between desire for higher vs. lower arousal and strategy use were observed.

**Table 3:** Repeated measures correlations between affective variables measured at time T−1 and emotion regulation strategy use in the subsequent inter-measurement interval measured at time T (N = 101; k = 1,386)

	Desire for arousal	Experienced HAP	Experienced LAP	Experienced LAN	Experienced HAN
Acceptance	.05	−.04	−.01	.08**	.06*
Cognitive reappraisal	.03	−.02	.02	−.00	−.01
Distraction	.06**	−.01	−.03	.07**	.04
Emotional support	.01	.07*	.02	−.06	−.01
Experiential suppression	.07*	−.05	−.09**	.06	.07**
Expressive suppression	.10**	−.05	−.12***	.05	.06
Planning	−.01	−.05	−.04	.04	.04
Relaxation	−.02	.07**	.08***	−.05*	−.03
Rumination	.05	−.11***	−.07*	.09**	.05

*Note*. * *p* < .05; ** *p* < .01; *** *p* < .001

Next, we constructed the full models predicting emotion regulation strategy use (measured at *T*) from desired high vs. low arousal (measured at *T*−1), adjusting for momentary experienced affect (HAP, LAP, LAN, and HAN measured at *T*−1). Parallel models were computed for each of nine specific emotion regulation strategies. We also constructed a baseline model in which any emotion regulation vs. no emotion regulation was predicted from desired arousal and experienced affect.

Both momentary experienced HAN (OR = 1.34, 95% CI = [1.06, 1.69], *p* = .01) and momentary desire for high vs. low arousal positive emotion (OR = 1.25, 95% CI = [1.08, 1.54], *p* = .005) were significant, positive predictors of *any* regulation, such that participants who reported experiencing higher HAN and ideally wanting more arousal (vs. less arousal) at time *T*−1 were more likely to report having engaged in at least one of the 9 emotion regulation strategies at time *T*. No statistically significant associations with experienced HAP (OR = 1.14, 95% CI = [.97, 1.13], *p* = .24), LAP (OR = 1.00, 95% CI = [.80, 1.26], *p* = .98), or LAN (OR = 1.11, 95% CI = [.88, 1.40], *p* = .38) were observed in this baseline model.

Having established this baseline for comparison, we next examined associations with specific emotion regulation strategies. As shown in Table 4, participants were more likely to report having engaged in distracting activities if they had previously reported feeling more unhappy or sluggish (i.e., higher experienced LAN) and to report having engaged in relaxing activities if they had previously reported feeling more calm or content (i.e., higher LAP). Conversely, participants were *less* likely to report having used expressive suppression if they had previously reported feeling higher LAP. Partly consistent with study hypotheses and consistent with zero-order correlations, desire for higher vs. lower arousal significantly predicted three specific strategies: distraction, experiential suppression, and expressive suppression, such that participants were more likely to say that they had used these strategies when they had previously endorsed a desire for higher arousal or energy.

**Table 4:** Time-lagged random-intercept mixed effects models predicting strategy likelihood at time T from actual arousal, ideal arousal, actual negative affect, and actual positive affect at T−1 (N = 101; k = 1,386)

		OR	95% CI	*p*
Acceptance				
	Desire for arousal (*T*−1)	1.15	.94, 1.41	.17
	Experienced HAP (*T*−1)	.91	.68, 1.20	.49
	Experienced LAP (*T*−1)	1.29	.98, 1.71	.07
	Experienced LAN (*T*−1)	1.29	.97, 1.71	.08
	Experienced HAN (*T*−1)	1.19	.91, 1.54	.21
Cognitive reappraisal				
	Desire for arousal (*T*−1)	1.09	.81, 1.46	.56
	Experienced HAP (*T*−1)	.83	.56, 1.24	.36
	Experienced LAP (*T*−1)	1.20	.81, 1.78	.35
	Experienced LAN (*T*−1)	.97	.65, 1.45	.90
	Experienced HAN (*T*−1)	1.02	.69, 1.50	.93
Distraction				
	Desire for arousal (*T*−1)	1.22	1.05, 1.41	.01
	Experienced HAP (*T*−1)	1.19	.96, 1.47	.11
	Experienced LAP (*T*−1)	.93	.75, 1.15	.48
	Experienced LAN (*T*−1)	1.29	1.04, 1.60	.02
	Experienced HAN (*T*−1)	1.02	.83, 1.26	.84
Emotional support				
	Desire for arousal (*T*−1)	1.08	.85, 1.38	.53
	Experienced HAP (*T*−1)	1.27	.93, 1.73	.13
	Experienced LAP (*T*−1)	.85	.62, 1.16	.31
	Experienced LAN (*T*−1)	.79	.56, 1.11	.17
	Experienced HAN (*T*−1)	1.07	.77, 1.49	.68
Experiential suppression				
	Desire for arousal (*T*−1)	1.26	1.05, 1.53	.02
	Experienced HAP (*T*−1)	1.09	.82, 1.44	.56
	Experienced LAP (*T*−1)	.76	.58, 1.01	.06
	Experienced LAN (*T*−1)	.95	.72, 1.25	.70
	Experienced HAN (*T*−1)	1.20	.94, 1.54	.15
Expressive suppression				
	Desire for arousal (*T*−1)	1.42	1.12, 1.81	.004
	Experienced HAP (*T*−1)	1.15	1.09, 1.82	.42
	Experienced LAP (*T*−1)	.61	.43, .85	.004
	Experienced LAN (*T*−1)	.89	.64, 1.24	.49
	Experienced HAN (*T*−1)	1.04	.77, 1.40	.80
Planning				
	Desire for arousal (*T*−1)	.97	.78, 1.20	.76
	Experienced HAP (*T*−1)	.91	.68, 1.23	.55
	Experienced LAP (*T*−1)	.93	.69, 1.26	.65
	Experienced LAN (*T*−1)	1.05	.78, 1.43	.74
	Experienced HAN (*T*−1)	1.04	.78, 1.39	.79
Relaxation				
	Desire for arousal (*T*−1)	.97	.83, 1.13	.67
	Experienced HAP (*T*−1)	1.11	.90, 1.37	.31
	Experienced LAP (*T*−1)	1.30	1.05, 1.61	.02
	Experienced LAN (*T*−1)	.96	.77, 1.20	.71
	Experienced HAN (*T*−1)	1.11	.89, 1.37	.35
Rumination				
	Desire for arousal (*T*−1)	1.08	.87, 1.34	.47
	Experienced HAP (*T*−1)	.73	.53, 1.00	.05
	Experienced LAP (*T*−1)	1.03	.75, 1.40	.87
	Experienced LAN (*T*−1)	1.20	.89, 1.60	.23
	Experienced HAN (*T*−1)	.99	.75, 1.30	.92

### Predicting Experienced Affect from Strategy Use

Finally, to complement our analyses predicting strategy use, we examined the outcomes associated with emotion regulation strategy use for momentary experienced HAP, LAP, LAN, and HAN. We first examined the zero-order repeated measures correlations. Consistent with expectations, distraction was associated with higher HAP, but not higher LAP in these analyses, as shown in Table 5. Partially consistent with expectations, relaxation was associated with higher LAP, but was also associated with higher HAP.

**Table 5:** Zero-order measures correlations between experienced affect at time T and emotion regulation strategy use in the preceding inter-measurement interval measured at time T (N = 101; k = 1,932)

	Experienced HAP	Experienced LAP	Experienced LAN	Experienced HAN
Acceptance	.02	.04	.03	.02
Cognitive reappraisal	.03	.03	.02	.02
Distraction	.08***	.04	−.01	−.04
Emotional support	.09***	.05*	−.01	.00
Experiential suppression	−.12***	−.12***	.11***	.12***
Expressive suppression	−.09***	−.12***	.12***	.10***
Planning	.06**	.00	−.02	.01
Relaxation	.17***	.22***	−.14***	−.15***
Rumination	−.13***	−.17***	.17***	.15***

*Note*. * *p* < .05; ** *p* < .01; *** *p* < .001

Next, we constructed the full models, in which all nine strategies were entered simultaneously, alongside momentary affect at the prior timepoint in order to examine change in affect from timepoint-to-timepoint. As shown in Table 6, emotional support, planning, and relaxation were significantly associated with increased HAP, whereas experiential suppression and rumination were associated with decreased HAP. Only one of the nine strategies, relaxation, was associated with increased LAP, consistent with study expectations, whereas experiential suppression, expressive suppression, and rumination were all associated with decreased LAP. Those same three strategies (i.e., experiential suppression, expressive suppression, and rumination) were also associated with higher LAN and higher HAN. Both planning and relaxation were associated with decreased LAN, but relaxation was unique in being associated with decreased HAN.

**Table 6:** Random-intercept mixed effects models predicting experienced affect at time T from prior emotion regulation strategy use (N = 101; k = 1,386)

	Experienced HAP			Experienced LAP			Experienced LAN			Experienced HAN		
	*b*	95% CI	*p*	*b*	95% CI	*p*	*b*	95% CI	*p*	*b*	95% CI	*p*
Experienced HAP (*T*−1)	.35	.30, .40	< .001	--	--	--	--	--	--	--	--	--
Experienced LAP (*T*−1)	--	--	--	.32	.28, .37	< .001	--	--	--	--	--	--
Experienced LAN (*T*−1)	--	--	--	--	--	--	.34	.29, .39	< .001	--	--	--
Experienced HAN (*T*−1)	--	--	--	--	--	--	--	--	--	.36	.31, .41	< .001
Acceptance	.02	−.09, .14	.70	.08	−.04, .20	.21	−.03	−.15, .08	.60	−.09	−.20, .03	.14
Cognitive reappraisal	−.01	−.19, .17	.92	.15	−.03, .34	.11	−.02	−.20, .16	.83	.00	−.17, .18	.97
Distraction	.07	−.02, .16	.11	−.01	−.10, .08	.85	.03	−.06, .12	.47	−.06	−.15, .03	.18
Emotional support	.23	.09, .36	.001	.14	−.00, .28	.05	−.09	−.23, .05	.19	.02	−.12, .16	.76
Experiential suppression	−.26	−.39, −.14	< .001	−.26	−.39, −.13	< .001	.21	.09, .33	.001	.19	.07, .31	.003
Expressive suppression	−.10	−.25, .05	.20	−.16	−.32, −.00	.05	.24	.09, .40	.002	.17	.02, .33	.03
Planning	.20	.07, .34	.003	.09	−.05, .23	.20	−.16	−.29, −.02	.02	−.06	−.20, .07	.37
Relaxation	.18	.08, .27	< .001	.37	.28, .47	< .001	−.24	−.33, −.15	< .001	−.30	−.40, −.21	< .001
Rumination	−.29	−.42, −.16	< .001	−.48	−.62, −.34	< .001	.39	.25, .52	< .001	.28	.15, .42	< .001

## Discussion

Parallel lines of research on emotion regulation use and choice, emotion regulation motives, and affect valuation converge on the conclusion that both our current emotional state and our desired emotional state shape our emotion regulation efforts. Consistent with prior research on need for regulation and hedonic motives, for example, higher momentary HAN was associated with greater use of any emotion regulation strategy in our data (e.g., Compas et al., 2017; Dixon-Gordon et al., 2015). To our knowledge, though, this is the first study to investigate whether and how the momentary desire to experience high vs. low arousal positive emotion influences spontaneous emotion regulation strategy use in daily life.

To establish a baseline for comparison, we first evaluated a model predicting use of *any* emotion regulation strategy to use of none of the listed emotion regulation strategies. In this baseline model, we found that participants were more likely to report regulating their emotions when they wanted to experience higher arousal or energy, even adjusting for experienced affect. Analyses of specific emotion regulation strategies, though, suggested that this effect was primarily driven by associations with three emotion regulation strategies that are prototypes of disengagement (Naragon-Gainey et al., 2017; Sheppes, 2020): distraction, experiential suppression, and expressive suppression. That is, when participants said that they wanted to feel more energized, they were more likely to report at the following timepoint that they had attempted to distract themselves with activity, control their emotional expressions, and ignore or suppress their feelings.

This finding is particularly intriguing given that complementary analyses suggested that none of these three disengagement strategies delivered sustained enhancement of HAP. In fact, participants who reported having used experiential suppression in the preceding hours tended to report diminished HAP and LAP (and increased LAN and HAN), and the same was true for rumination, potentially suggesting that these two strategies exert a stronger influence on the valence continuum than on arousal. Use of expressive suppression predicted lower LAP and higher LAN and HAN, but was not significantly associated with HAP. These results may explain, in part, why desire for higher arousal was not systematically associated with increased HAP at the subsequent timepoint. Meanwhile, consistent with study hypotheses, relaxation was unique in that it was the only strategy associated with higher LAP—complementarily, it was also the only strategy associated with lower HAN—although it was also associated with higher HAP. Use of emotional support and planning predicted gains specifically in HAP (i.e., they were not associated with LAP). Planning was also associated with decreased LAN, potentially suggesting that planning may be the strategy that is most well-suited specifically to increasing positively valenced arousal. Contrary to study expectations, distraction, as well as acceptance and reappraisal, was not associated with momentary affect when adjusting for other emotion regulation strategies and affect at the prior timepoint. That distraction was not systematically associated with changes in experienced affect may speak to the flexibility of this strategy in that the content of distracting activities can vary widely and in that a given activity can have different consequences depending on one’s baseline (e.g., going for a run can be activating in the context of boredom or calming in the context of intense arousal). Although extant EMA research on high and low arousal positive affective consequences of specific emotion regulation strategies is limited (for review, see Colombo et al., 2020), our findings in this regard largely align with prior findings of decreased positive affect following use of disengagement strategies, such as experiential avoidance (Brans et al., 2013; Heiy & Cheavens, 2014), expressive suppression (Brans et al., 2013; but see also Heiy & Cheavens, 2014), and rumination (Brans et al., 2013; Li et al., 2017), as well as increased positive affect following problem-solving and social support (Heiy & Cheavens, 2014).

Taken together, our data suggest that desire for higher energy and arousal forecasts higher likelihood of disengagement strategies in the following hours, notwithstanding that these strategies do not appear to be effective for enhancing HAP in that same timeframe. To the contrary, the strategies that did predict sustained enhancement of HAP (i.e., relaxation, emotional support, planning) align more with behavioral engagement (cf. Naragon-Gainey et al., 2017). In essence, then, these data point to an apparent disconnect between the strategies that our participants tended to use when they wanted to feel more energized and the subsequent affective outcomes associated with use of those strategies.

Beyond these findings related to emotion regulation, we also found evidence for considerable within-person variation in momentary desire for high vs. low arousal positive emotion. Examination of the 95% confidence intervals for the intraclass correlations suggested that there was comparatively more within-person variation in momentary desire for high vs. low arousal than in momentary experienced affect. Indeed, the predominance of the observed variance in desire for arousal reflected within-person fluctuations as opposed to between-person differences. In other words, while there are important between-person and between-culture differences in the extent to which people ideally prefer to feel more energized vs. more calm (Tsai, 2007), contextual factors play a critical role. These fluctuations, moreover, could not be immediately explained as a time-of-day effect, although we lacked sleep-wake and chronotype data needed to more fully investigate potential circadian influences.

As with all studies, the present investigation had notable strengths and limitations. There is considerable between- *and* within-person variability in emotional experience and emotion regulation strategy use, which highlights the need for intensive repeated measures designs such as the one used in the current study. Whereas we focused on within-person effects and the total number of observations for the time-lagged analyses was reasonably high (*k* = 1,386), the level-2 sample size was modest (*N* = 101). This sample, moreover, consisted of college students who endorsed recent engagement in risky behaviors, so it is plausible that this sample may evince a relatively high level of trait-like emotion dysregulation (cf. Swerdlow et al., 2020). Future work should examine the generalizability of these findings across samples characterized by varying prevalence of clinical behaviors.

There are some limitations in this study related to the self-reported measurement of affect. One concern is that prompting participants to report on discrepancies between their actual and ideal affect and on use of specific emotion regulation strategies may have made these more salient and thus altered their subsequent behavior. Another concern is that our momentary experienced affect composites were inter-correlated, and so shared predictive variance may have been an issue. Of note in this regard, associations between experienced affect and subsequent emotion regulation strategy use that were significant in zero-order analyses were frequently reduced to non-significance in the full models that simultaneously included all four quadrants of the affective circumplex. Further work should consider how to separate arousal and valence as cleanly as possible, perhaps by incorporating psychophysiological measures. Third, to minimize participant burden we used a single bipolar item for desire for arousal and only two items for each affect quadrant. Fourth, in endorsing a desire for higher arousal (or lower arousal), some participants may have been more motivated by a desire to reduce LAN (or HAN) than to increase HAP (or LAP), and this may have guided their choice of emotion regulation strategy. Similarly, it was not possible to distinguish a motivation to increase HAP versus decrease LAP. Future work could consider separately assessing desire for each quadrant and directly comparing overlap in these measurements. Future studies would also do well to consider the presence of interactions (e.g., between experienced and desired affect), which we were not adequately powered to test.

Another notable strength of the current study is that we were able to conduct time-lagged analyses, which allowed us to discern temporal precedence. Nonetheless, sampling frequency is a tricky consideration. We sampled participants, on average, every 2.3 h. The initial affective consequences of emotion regulation strategy use, though, may unfold within seconds or minutes in some cases. This is relevant to interpreting the apparent disconnect between goals and outcomes associated with experiential suppression. One possibility is that people may hope or believe that suppressing negative emotions will produce energetic gains, whereas, in reality, experiential suppression may be energy-depleting. A different possibility, though, is that experiential suppression allows people to conserve energy in the moment (e.g., by being easier to implement; cf. Milyavsky et al., 2019) but tends to lead to diminished energy down the line. Yet another possibility is that people may frequently overshoot the mark when they are aiming for higher arousal and then use experiential suppression to temper over-arousal. Sampling at a higher frequency would permit a finer-grained test of these contrasting interpretations.

Consistent with calls to study a broad range of emotion regulation studies, we examined 9 different strategies. We found evidence consistent with polyregulation, in that many of these strategies were positively correlated with one another (cf. Ford et al., 2019). At the same time, we also found considerable divergence in the predictors and outcomes associated with these strategies, again consistent with prior research. We did not, though, have a priori hypotheses about all of the nine strategies, and, to our knowledge, the observed associations between desire for arousal and emotion regulation strategy use are novel. These associations were, moreover, modest in magnitude. While we note that they were comparable in magnitude to the predictive effect of experienced affect on emotion regulation strategy use, correction for multiple comparison would render the associations between desire for arousal and distraction and between desire for arousal and experiential suppression non-significant. In other words, while we see these results as intriguing evidence of a connection between desire for arousal and use of disengagement strategies, these links should be replicated and further explored in confirmatory studies.

In conclusion, our data align with the idea that people’s momentary emotion goals—specifically, their arousal goals—shape their emotion regulation efforts above and beyond their experienced affect, consistent with an emerging emphasis on emotion and emotion regulation goals. Specifically, desire for higher arousal seemed to set the stage for greater use of disengagement strategies in our sample. At the same time, our data points to a possible disconnect between people’s stated emotion goals and the outcomes associated with their emotion regulation strategy use. In other words, this study was a first step in understanding which emotion regulation strategies people use in daily life to meet specific arousal goals—and which strategies successfully regulate in the desired direction. Given the variety of clinical concerns centered on arousal dysregulation and the apparent disconnects we observed, arousal regulation may be a particularly tricky dimension of emotion regulation. Further research should focus on which emotion regulation strategies can most help people regulate arousal in given contexts and effective ways to teach these skills in interventions. We hope that these findings will spur additional research aimed at understanding the behavioral and regulatory consequences associated with momentary emotion goals, particularly related to desire for high versus low arousal, and at unpacking the causes of the disconnects we observed.

## Footnotes

1 This expectation was supported by pilot data collected by one of the first authors (B. Swerdlow) as part of a pair of prior studies in undergraduate samples. First, in an autobiographical recall study of receiving intrinsic interpersonal emotion regulation (*N* = 371), only 1.9% of respondents indicated that their primary affective goal was to feel more negative emotion. Second, in a daily diary study of intra- and inter-personal emotion regulation (*N* = 86, *k* = 1,156), respondents indicated that only 2.8% of emotion regulation instances were associated with any desire to feel more negative emotion. See also Riediger et al., 2009.

## Additional Information

### Data Availability

Deidentified data are available on the OSF at https://osf.io/htpqk/?view_only=9a45dd4926e04161b3cd31aa1c31c242.

### Code Availability

R code used for data analyses are available on the OSF at https://osf.io/htpqk/?view_only=9a45dd4926e04161b3cd31aa1c31c242.

## References

[CR1] Aldao A, Nolen-Hoeksema S, Schweizer S (2010). Emotion-regulation strategies across psychopathology: A meta-analytic review. Clinical Psychology Review.

[CR2] Aldao A, Sheppes G, Gross JJ (2015). Emotion regulation flexibility. Cognitive Therapy and Research.

[CR3] Bakdash JZ, Marusich LR (2017). Repeated measures correlation. Frontiers in Psychology.

[CR4] Bartoń, K. (2009). MuMIn: Multi-model inference.

[CR5] Barrett L. F. (2004). Feelings or words? Understanding the content in self-report ratings of experienced emotion. *Journal of Personality and Social Psychology*, *87*(2), 266–281. 10.1037/0022-3514.87.2.266 10.1037/0022-3514.87.2.266 PMC1351136 15301632

[CR6] Bates D, Mächler M, Bolker B, Walker S (2015). Fitting linear mixed-effects models using lme4. Journal of Statistical Software.

[CR7] Bloch L, Haase CM, Levenson RW (2014). Emotion regulation predicts marital satisfaction: More than a wives’ tale. Emotion.

[CR8] Bonanno GA, Burton CL (2013). Regulatory flexibility: An individual differences perspective on coping and emotion regulation. Perspectives on Psychological Science.

[CR9] Brans K, Koval P, Verduyn P, Lim YL, Kuppens P (2013). The regulation of negative and positive affect in daily life. Emotion.

[CR10] Carver CS, Scheier MF (2001). *On the self-regulation of behavior*.

[CR11] Catterson AD, Eldesouky L, John OP (2017). An experience sampling approach to emotion regulation: Situational suppression use and social hierarchy. Journal of Research in Personality.

[CR12] Chim L, Hogan CL, Fung HH, Tsai JL (2018). Valuing calm enhances enjoyment of calming (vs. exciting) amusement park rides and exercise. Emotion.

[CR13] Colombo D, Fernández-Álvarez J, Suso-Ribera C, Cipresso P, Valev H, Leufkens T, Sas C, Garcia-Palacios A, Riva G, Botella C (2020). The need for change: Understanding emotion regulation antecedents and consequences using ecological momentary assessment. Emotion.

[CR14] Compas BE, Jaser SS, Bettis AH, Watson KH, Gruhn MA, Dunbar JP (2017). Coping, emotion regulation, and psychopathology in childhood and adolescence: A meta-analysis and narrative review. Psychological Bulletin.

[CR15] Conrad A, Roth WT (2007). Muscle relaxation therapy for anxiety disorders: It works but how?. Journal of Anxiety Disorders.

[CR16] Dixon-Gordon KL, Aldao A, De Los Reyes A (2015). Emotion regulation in context: Examining the spontaneous use of strategies across emotional intensity and type of emotion. Personality and Individual Differences.

[CR17] Eldesouky L, English T (2019). Regulating for a reason: Emotion regulation goals are linked to spontaneous strategy use. Journal of Personality.

[CR18] Eldesouky L, Gross JJ (2019). Emotion regulation goals: An individual difference perspective. Social and Personality Psychology Compass.

[CR19] Fischer, A. H., Manstead, A. S. R., Evers, C., Timmers, M., & Valk, G. (2004). *Motives and norms underlying emotion regulation.* In P. Philippot & R. S. Feldman (Eds.), *The regulation of emotion* (p. 187–210). Lawrence Erlbaum Associates Publishers.

[CR20] Ford BQ, Gross JJ, Gruber J (2019). Broadening our field of view: The role of emotion polyregulation. Emotion Review.

[CR21] Gross JJ (2015). Emotion regulation: Current status and future prospects. Psychological Inquiry.

[CR22] Halekoh U, Højsgaard S (2014). A Kenward-Roger approximation and parametric bootstrap methods for tests in linear mixed models—The R package pbkrtest. Journal of Statistical Software.

[CR23] Heiy JE, Cheavens JS (2014). Back to basics: A naturalistic assessment of the experience and regulation of emotion. Emotion.

[CR24] Hopko DR, Lejuez CW, Ruggiero KJ, Eifert GH (2003). Contemporary behavioral activation treatments for depression: Procedures, principles, and progress. Clinical Psychology Review.

[CR25] Hopper SI, Murray SL, Ferrara LR, Singleton JK (2019). Effectiveness of diaphragmatic breathing for reducing physiological and psychological stress in adults: A quantitative systematic review. JBI Evidence Synthesis.

[CR26] Kenward MG, Rogers JH (1997). Small sample inference for fixed effects from restricted maximum likelihood. Biometrics.

[CR27] Kline, R. B. (2011). *Principles and practice of structural equation modeling* (3rd ed.). Guilford Press.

[CR28] Kuznetsova A, Brockhoff PB, Christensen RH (2017). lmerTest package: Tests in linear mixed effects models. Journal of Statistical Software.

[CR29] Lennarz HK, Hollenstein T, Lichtwarck-Aschoff A, Kuntsche E, Granic I (2019). Emotion regulation in action: Use, selection, and success of emotion regulation in adolescents’ daily lives. International Journal of Behavioral Development.

[CR30] Li YI, Starr LR, Hershenberg R (2017). Responses to positive affect in daily life: Positive rumination and dampening moderate the association between daily events and depressive symptoms. Journal of Psychopathology and Behavioral Assessment.

[CR31] Lüdecke, D. (2021). *sjstats: Statistical Functions for Regression Models (Version 0.18.1)*. 10.5281/zenodo.1284472

[CR32] Lüdecke, D., Ben-Shachar, M., Patil, I., Waggoner, P., & Makowski, D. (2021). Performance: An R package for assessment, comparison and testing of statistical models. *Journal of Open Source Software*, (6, 60), 3139. 10.21105/joss.03139

[CR33] Mauss IB, Tamir M (2014). *Emotion goals: How their content, structure, and operation shape emotion regulation*.

[CR34] McMahon TP, Naragon-Gainey K (2019). The multilevel structure of daily emotion-regulation-strategy use: An examination of within-and between-person associations in naturalistic settings. Clinical Psychological Science.

[CR35] Millgram Y, Sheppes G, Kalokerinos EK, Kuppens P, Tamir M (2019). Do the ends dictate the means in emotion regulation?. Journal of Experimental Psychology: General.

[CR36] Milyavsky M, Webber D, Fernandez JR, Kruglanski AW, Goldenberg A, Suri G, Gross JJ (2019). To reappraise or not to reappraise? Emotion regulation choice and cognitive energetics. Emotion.

[CR37] Moreno PI, Moskowitz AL, Ganz PA, Bower JE (2016). Positive affect and inflammatory activity in breast cancer survivors: Examining the role of affective arousal. Psychosomatic Medicine.

[CR38] Nakagawa S, Schielzeth H (2013). A general and simple method for obtaining R2 from generalized linear mixed-effects models. Methods in Ecology and Evolution.

[CR39] Naragon-Gainey K, McMahon TP, Chacko TP (2017). The structure of common emotion regulation strategies: A meta-analytic examination. Psychological Bulletin.

[CR40] Operario, D., Adler, N. E., & Williams, D. R. (2004). Subjective social status: Predictive utility for global health. *Psychology and Health*, *19*(2), 237–246. 10.1080/08870440310001638098

[CR41] Opitz PC, Gross JJ, Urry HL (2012). Selection, optimization, and compensation in the domain of emotion regulation: Applications to adolescence, older age, and major depressive disorder. Social and Personality Psychology Compass.

[CR42] Painter JM, Mote J, Peckham AD, Lee EH, Campellone TR, Pearlstein JG, Morgan S, Kring AM, Johnson SL, Moskowitz JT (2019). A positive emotion regulation intervention for bipolar I disorder: Treatment development and initial outcomes. General Hospital Psychiatry.

[CR43] Posner J, Russell JA, Peterson BS (2005). The circumplex model of affect: An integrative approach to affective neuroscience, cognitive development, and psychopathology. Development and Psychopathology.

[CR44] R Core Team (2020). R: A language and environment for statistical computing. R Foundation for Statistical Computing, Vienna, Austria. https://www.R-project.org/

[CR45] Riediger M, Schmiedek F, Wagner GG, Lindenberger U (2009). Seeking pleasure and seeking pain: Differences in prohedonic and contra-hedonic motivation from adolescence to old age. Psychological Science.

[CR46] Russell JA (1980). A circumplex model of affect. Journal of Personality and Social Psychology.

[CR47] Scheibe S, English T, Tsai JL, Carstensen LL (2013). Striving to feel good: Ideal affect, actual affect, and their correspondence across adulthood. Psychology and Aging.

[CR48] Sheppes G, Gawronksi B (2020). Transcending the “good & bad” and “here & now” in emotion regulation: Costs and benefits of strategies across regulatory stages. *Advances in experimental social psychology (Vol. 61, pp. 185-236)*.

[CR49] Sheppes G, Scheibe S, Suri G, Gross JJ (2011). Emotion-regulation choice. Psychological Science.

[CR50] Sheppes G, Scheibe S, Suri G, Radu P, Blechert J, Gross JJ (2014). Emotion regulation choice: A conceptual framework and supporting evidence. Journal of Experimental Psychology: General.

[CR51] Sims T, Tsai JL, Koopmann-Holm B, Thomas EA, Goldstein MK (2014). Choosing a physician depends on how you want to feel: The role of ideal affect in health-related decision making. Emotion.

[CR52] Southward MW, Cheavens JS (2020). More (of the right strategies) is better: Disaggregating the naturalistic between-and within-person structure and effects of emotion regulation strategies. Cognition and Emotion.

[CR53] Swerdlow BA, Pearlstein JG, Sandel DB, Mauss IB, Johnson SL (2020). Maladaptive behavior and affect regulation: A functionalist perspective. Emotion.

[CR54] Tamir M (2016). Why do people regulate their emotions? A taxonomy of motives in emotion regulation. Personality and Social Psychology Review.

[CR55] Troy AS, Shallcross AJ, Mauss IB (2013). A person-by-situation approach to emotion regulation: Cognitive reappraisal can either help or hurt, depending on the context. Psychological Science.

[CR56] Tsai JL (2007). Ideal affect: Cultural causes and behavioral consequences. Perspectives on Psychological Science.

[CR57] Tsai JL (2017). Ideal affect in daily life: Implications for affective experience, health, and social behavior. Current Opinion in Psychology.

[CR58] Wang L, Zhang Q, Maxwell SE, Bergeman CS (2019). On standardizing within-person effects: Potential problems of global standardization. Multivariate Behavioral Research.

[CR59] Wenzel, M., Rowland, Z., Weber, H., & Kubiak T. (2020). A round peg in a square hole: Strategy-situation fit of intra- and interpersonal emotion regulation strategies and controllability. *Cognition and Emotion*, *34*(5), 1003–1009. 10.1080/02699931.2019.1697209 10.1080/02699931.2019.1697209 31790333

[CR60] Wilms R, Lanwehr R, Kastenmüller A (2020). Emotion regulation in everyday life: The role of goals and situational factors. Frontiers in Psychology.

[CR61] Young G, Suri G (2020). Emotion regulation choice: A broad examination of external factors. Cognition and Emotion.

[CR62] Zuur AF, Ieno EN, Elphick CS (2010). A protocol for data exploration to avoid common statistical problems. Methods in Ecology and Evolution.

